# Supplementary figures and images for: Genome-Wide Analysis of the SPL Gene Family and Expression Analysis during Flowering Induction in Prunus × yedoensis ‘Somei-yoshino’
Source: Int J Mol Sci. 2022 Sep 2;23(17):10052. doi: 10.3390/ijms231710052 (PMC9456211; doi:10.3390/ijms231710052)

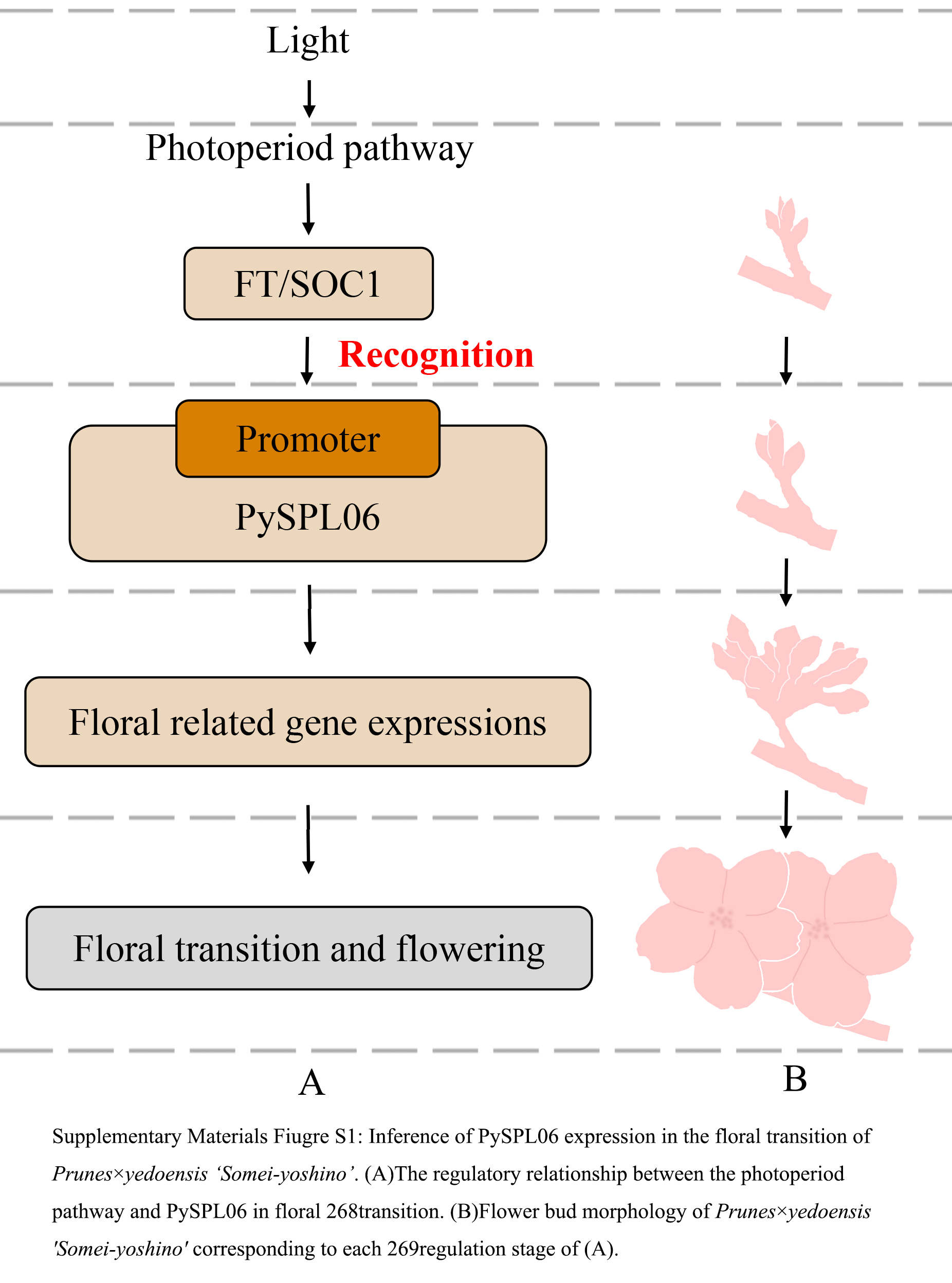

Supplement: Supplementary file 1 [file ijms-23-10052-s001.zip › Supplementary Materials Figure S1 Inference of PySPL06 expression in the floral transition.jpg]
